# Starch Branching Enzyme 1 Is Important for Amylopectin Synthesis and Cyst Reactivation in Toxoplasma gondii

**DOI:** 10.1128/spectrum.01891-21

**Published:** 2022-04-21

**Authors:** Jing Yang, Zhengming He, Chengjie Chen, Junlong Zhao, Rui Fang

**Affiliations:** a State Key Laboratory of Agricultural Microbiology, College of Veterinary Medicine, Huazhong Agricultural University, Wuhan, Hubei, China; b College of Veterinary Medicine, Yunnan Agricultural University, Kunming, Yunnan, China; Weill Cornell Medicine

**Keywords:** *Toxoplasma gondii*, amylopectin synthesis, starch branching enzyme, bradyzoites, reactivation

## Abstract

Toxoplasma gondii (T. gondii) bradyzoites facilitate chronic infections that evade host immune response. Furthermore, reactivation in immunocompromised individuals causes severe toxoplasmosis. The presence of abundant granules containing the branched starch amylopectin is major characteristic of bradyzoites that is nearly absent from tachyzoites that drive acute disease. T. gondii genome encodes to potential Starch branching enzyme 1 (SBE1) that creates branching during amylopectin biosynthesis. However, the physiological function of the amylopectin in T. gondii remains unclear. In this study, we generated a *SBE1* knockout parasites and revealed that deletion of *SBE1* caused amylopectin synthesis defects while having no significant impact on the growth of tachyzoites under normal culture conditions *in vitro* as well as virulence and brain cyst formation. Nevertheless, *SBE1* knockout decreased the influx of exogenous glucose and reduced tachyzoites proliferation in nutrition-deficient conditions. Deletion of *SBE1* together with the α-amylase (α-AMY), responsible for starch digestion, abolished amylopectin production and attenuated virulence while restoring brain cyst formation. In addition, cysts with defective amylopectin metabolism showed abnormal morphology and were avirulent to mice. In conclusion, SBE1 is essential for the synthesis of amylopectin, which serves as energy storage during the development and reactivation of bradyzoites.

**IMPORTANCE** Toxoplasmosis has become a global, serious public health problem due to the extensiveness of the host. There are great differences in the energy metabolism in the different stages of infection. The most typical difference is the abundant accumulation of amylopectin granules in bradyzoites, which is almost absent in tachyzoites. Until now, the physiological functions of amylopectin have not been clearly elucidated. We focused on starch branching enzyme 1 (SBE1) in the synthesis pathway to reveal the exact physiological significance of amylopectin. Our study clarified the role of SBE1 in the synthesis pathway and amylopectin in tachyzoites and bradyzoites, and demonstrated that amylopectin, as an important carbon source, was critical to parasites growth under an unfavorable environment and the reactivation of bradyzoites to tachyzoites. The findings obtained from our study provides a new avenue for the development of *Toxoplasma* vaccines and anti-chronic toxoplasmosis drugs.

## INTRODUCTION

Toxoplasma gondii (T. gondii) infection in immunocompromised patients can result in encephalitis or pneumonia, and even death (1, 2). The infection has three characteristic stages, including tachyzoites, bradyzoites and sporulated oocysts, with tachyzoites replicating rapidly during acute infection. Although immunocompetent individuals can eventually restrain tachyzoites proliferation, conversion to bradyzoites causes chronic infections that escape host immune response (3–5). The transformation between tachyzoites and bradyzoites involves changes in morphology and stage-specific protein expression and metabolism, which is crucial for the transmission and pathogenicity of T. gondii among different hosts (6–8). T. gondii can store glucose in the form of amylopectin in a similar manner to some photosynthetic microorganisms (9). Massive accumulation of cytoplasmic granules containing the branched starch amylopectin is striking feature of bradyzoites that is nearly absent from tachyzoites that drive acute disease (10, 11). Amylopectin has been shown to play a vital role several coccidian parasites. For instance, decreased amylopectin levels in *Eimeria* oocysts have been associated with reduced vitality and infectivity of oocysts (12). In addition, T. gondii sporozoites are unable to acquire nutriment from the environment; hence, *in vivo* starch granules may be an important energy source (13).

The amylopectin synthesis pathway in T. gondii has been comprehensively elucidated (14, 15). First, glucose phosphomutase catalyzes glucose-6-phosphate into glucose-1-phosphate, in turn, catalyzed by UDP-glucose pyrophosphorylase to generate UDP-glucose. UDP-glucose is then synthesized into short-chain glucans. These glucans can then be extended by linear polymers by starch synthase through the addition glucose units with α-1,4 glycosidic bonds. Finally, starch branching enzyme (SBE) can create new branches off these linear polymers via the addition of glucose α-1,6-glycosidic bond. Amylopectic digestion begins with phosphorylation by α-glucan, water dikinase phosphorylates glucose, upon which α-amylase cleaves the α-1,4-glycosidic bond to generate glucose and maltose. Starch debranching enzymes hydrolyze the branched α-1,6 linkages, and finally, α-1,4-glycosidic chains are hydrolyzed by glycogen phosphorylase (GP) to release glucose-1-phosphate molecules. Recent studies have indicated that Ca^2+^-dependent protein kinase 2 (TgCDPK2) as a key regulator of amylopectin metabolism. It can phosphorylate enzymes involved in amylopectin metabolism nad genetic ablation of *CDPK2* leads to the absence of visible cyst formation (16). Therefore, amylopectin accumulation may be a determining factor of cyst development. For instance, the number of brain cysts is significantly decreased in infected patients with glycogen phosphorylase (GP) S25 phosphorylated mutants, indicating that the storage and utilization of amylopectin require GP phosphorylation as an indispensable part of chronic infection maintenance (14). Furthermore, lack of starch synthase (SS), essential for amylopectin synthesis, reduces amylopectin production, tachyzoites proliferation, and impairs the reactivation of bradyzoites to tachyzoites (15). It has been proposed that amylopectin may provide energy for the conversion of bradyzoites to tachyzoites; however, this hypothesis has not been clearly illustrated. Therefore, it is imperative to gain a deeper understanding of the physiological functions of amylopectin to prevent and control toxoplasmosis.

SBE1 is one of the primary enzymes involved in amylopectin synthesis in plants and algae. Its activity determines the structure and physical properties of amylopectin granules (17). Furthermore, it is responsible for enzymatic hydrolysis of α-1,4-glycosidic bonds from the donor chain, and transfers the hydrolyzed chain to the acceptor chain to form branches containing α-1,6-glycosidic bonds (18). It has been shown that downregulated expression of SBE1 in corn resulted in the expected decrease in branched amylopectin and corresponding increase in the linear amylose polymers (19). Two putative genes encoding SBEs (*TGME49_316520* and *TGME49_209960*) exist in the T. gondii genome. Nevertheless, at present, our understanding of the biological function of SBEs in the amylopectin metabolism of T. gondii remains limited. Here, we focused on the SBE1-encoding gene (*TGME49_316520*) and show that it contributes to amylopectin biosynthesis and is involved in the regulatory mechanism of bradyzoite reactivation.

## RESULTS

### *Toxoplasma* amylopectin synthesis requires SBE1.

**FIG 1:**
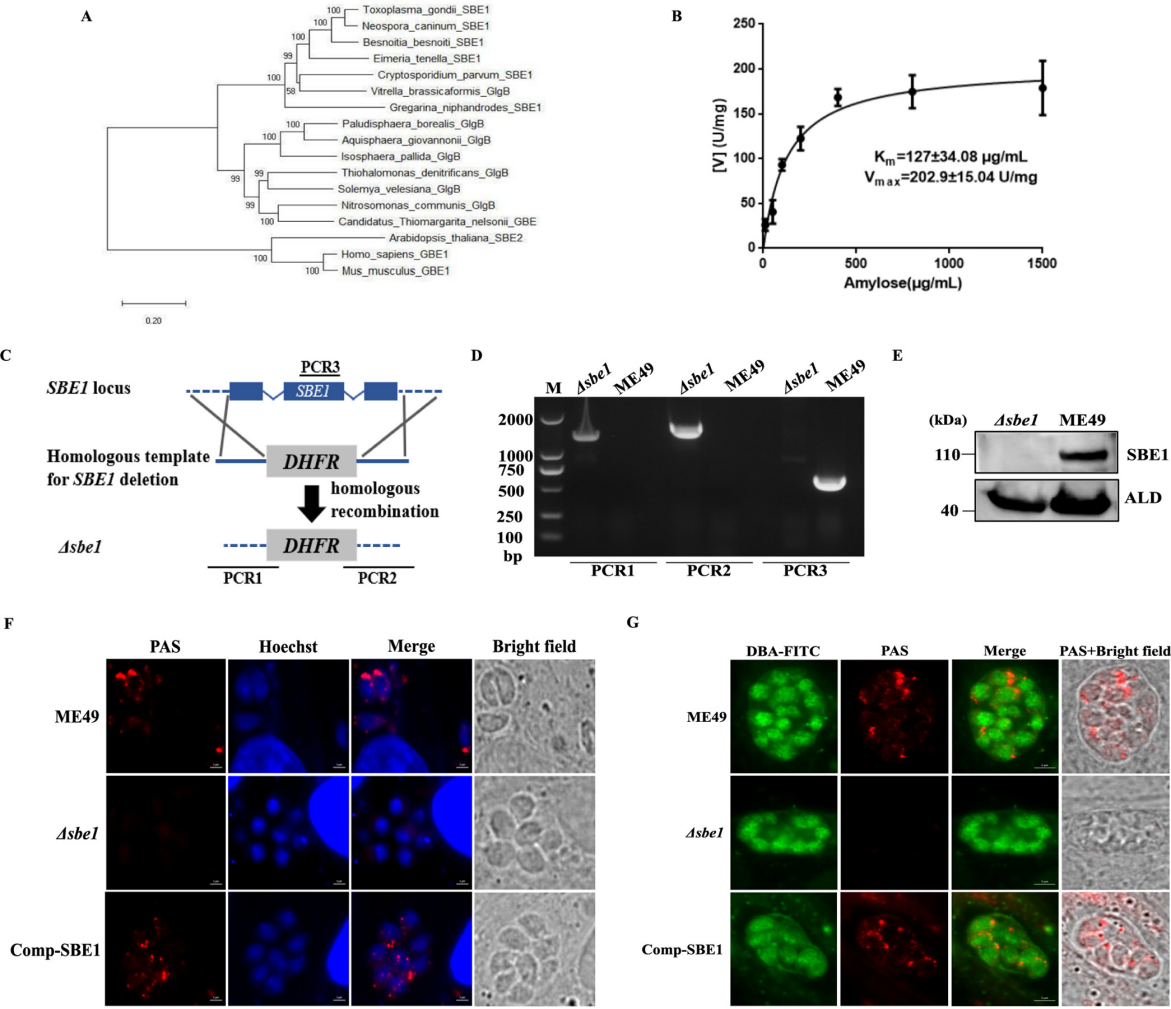
Knockout of *SBE1* results in amylopectin synthesis defects in the tachyzoites and bradyzoites. A. Evolution relationship analysis of starch branching enzymes from Neospora caninum, Besnoitia besnoiti, *Eimeria tenella*, Vitrella brassicaformis, Cryptosporidium parvum, *Gregarina niphandrodes*, *Arabidopsis*, Thiohalomonas denitrificans, *Solemya velesiana*, Nitrosomonas communis, *Candidatus Thiomargarita nelsonii*, Isosphaera pallida, Paludisphaera borealis, Aquisphaera giovannonii, Homo sapiens, Mus musculus, and T. gondii. Protein sequences were searched from the Uniprot database and the phylogenetic tree was constructed using the maximum-likelihood method (MEGA7). B. Detection of enzyme kinetics of recombinant protein starch branching enzyme 1 (SBE1). C. Schematic illustration of generation of the *Δsb1e* mutant via CRISPR/CAS9-mediated technique. D. Diagnostic PCRs on a Δsbe1: DHFR* clone. E. Western blotting confirmed disruption of SBE1 in transgenic parasites. Parasite lysates were incubated with mouse anti-SBE1 antibody, while rabbit anti-ALD served as the loading control. F. Amylopectin accumulation in tachyzoites detected with PAS staining. Parasites were inoculated to HFF host cells and incubated for 24 h under normal culture conditions for tachyzoites. G. Amylopectin accumulation in bradyzoites detected with PAS staining. Parasites were inoculated to HFF host cells and incubated under conditions of pH 8.2 medium without CO_2_ for 4 days in bradyzoites.

To investigate the role of amylopectin in T. gondii, we focused on *Tg*SBE1, a protein encoded by *TGME49_316520*, which showed higher expression than *Tg*SBE2 (*TGME49_209960*) in the bradyzoites based on the information obtained from the ToxoDB database, thus we speculated that *Tg*SBE1 may play a more important role in bradyzoites. The amino acid sequence of *Tg*SBE1 was relatively conserved in the apicomplexans Neospora caninum, Besnoitia besnoiti, *Eimeria tenella* and Cryptosporidium parvum (Fig. S1 in the supplemental material). Furthermore, *Tg*SBE1 was more closely related to SBEs in bacteria and plant from phylogenetic analysis (Fig. 1A).

When amylose was used as the reaction substrate to measure SBE1 activity, it revealed that recombinant TgSBE1 possessed the activity of catalyzing amylose to amylopectin synthesis, with *K*_m_ and *V*_max_ of 127 ± 34.08 μg/mL and 202.9 ± 15.04 U/mg, respectively (Fig. 1B).

To confirm the function of SBE1 in amylopectin synthesis, CRISPR/Cas9-mediated homologous recombination technology used to knock out the *SBE1* gene in the ME49 strain as verified by PCR and Western blotting (Fig. 1C–E). Upon PAS staining of amylopectin (Fig. 1F–G), we found that tachyzoites showed weak staining in the wild-type parent, whereas no visible PAS signal appeared in the *Δsbe1* mutant, with similar observations made under alkaline-induced bradyzoite culture conditions. To further verify that the amylopectin synthesis defect was indeed caused by the deletion of *SBE1*, we subsequently complemented the knockout by inserting the C-terminally Ty1-tagged SBE1 coding sequence under the control of the alpha-tubulin promoter into the locus of *HXGPRT* in the *Δsbe1* mutant (Fig. S2A in the supplemental material). The PCR screening and immunostaining confirmed the integration of the complementing constructs and expression of the protein (Fig. S2B–C). Meanwhile, amylopectin synthesis defects were authentically rescued in both tachyzoites and bradyzoites of the Comp-SBE1 strain (Fig. 1F–G). The above results confirmed that SBE1 was indispensable for amylopectin synthesis in T. gondii.

### Loss of SBE1 impairs the intracellular replication of tachyzoites *in vitro* under nutrition-deficient culture conditions.

**FIG 2:**
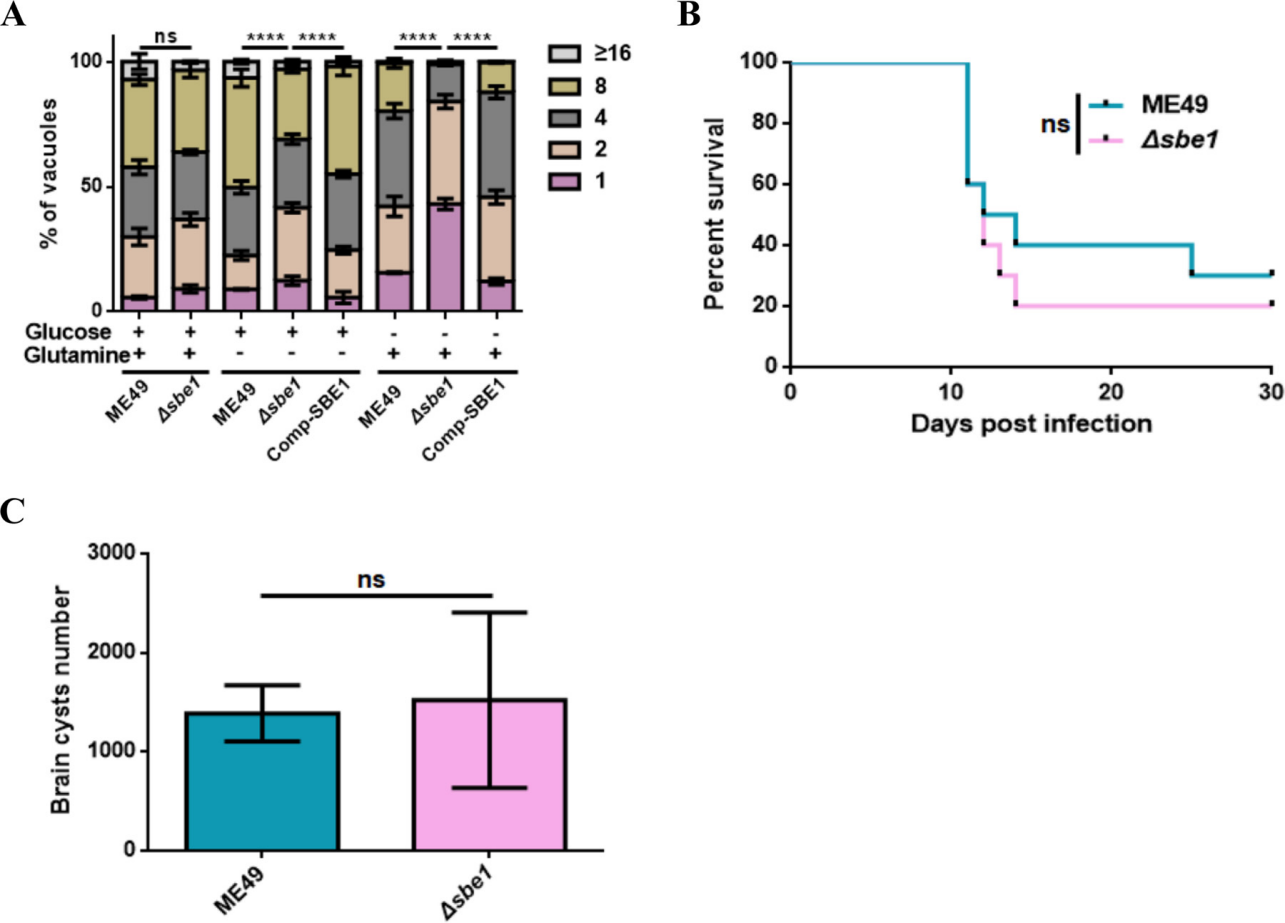
Intracellular replication and virulence tests of *Δsbe1* mutant. A. The effect of *SBE1* deletion on the replication ability of tachyzoites in different carbon source mediums, and intracellular replication rates were determined by the fraction of vacuoles containing 1, 2, 4, 8, and 16 or more parasites. B. Survival curves of mice infected with tachyzoites of indicated strains. ME49 and *Δsbe1* strains were respectively used to infect ICR mice (100 parasites per mouse, 10 mice for each group) by intraperitoneal injection, and the survival of mice was monitored for 30 days. C. Brain cysts of ICR mice infected with corresponding strains. The brain cysts of positive mice that survived 30 days after infection with the indicated strains were collected and stained with DBA-FITC, and the number of brain cysts was counted under a microscope. ****, P < 0.0001, ns represents not significant, two-way ANOVA, Mantel-Cox log-rank test. Data represents mean ± SEM of three independent experiments.

To explore the function of SBE1 in tachyzoite growth, we conducted a tachyzoite replication assay and found no significant difference of tachyzoite replication between the wild-type strain and *SBE1* deletion mutant under standard culture conditions (Fig. 2A). Considering that both glucose and glutamine can be used as important carbon sources to provide energy for the parasite growth *in vitro* (20), and we next determined the replication ability of tachyzoites in medium only with one carbon source. The results showed that the replication degree of the *Δsbe1* mutant was significantly reduced in the absence of glutamine (Fig. 2A), which implied that the demand for glutamine increased after SBE1 inactivation. In a medium containing glutamine but no glucose, the replication ability was even further diminished, which indicated that tachyzoite growth under nutrition-deficient culture conditions requires SBE1. Moreover, these results also further indicated that amylopectin can be used as a vital carbon source to provide energy for parasite growth *in vitro*.

Nevertheless, the results of the virulence assay revealed that loss of SBE1 in tachyzoites still showed strong virulence in mice, which was consistent with the growth ability *in vitro* (Fig. 2B). It has been reported that deletion of amylopectin metabolic pathway enzymes has no effect on tachyzoite growth, yet may reduce brain cyst formation (14, 21). However, the result was beyond expectation that the absence of SBE1 has almost no influence on brain cyst formation (Fig. 2C), and is therefore not essential for virulence and brain cyst formation.

### Loss of SBE1 causes metabolic alteration.

**FIG 3:**
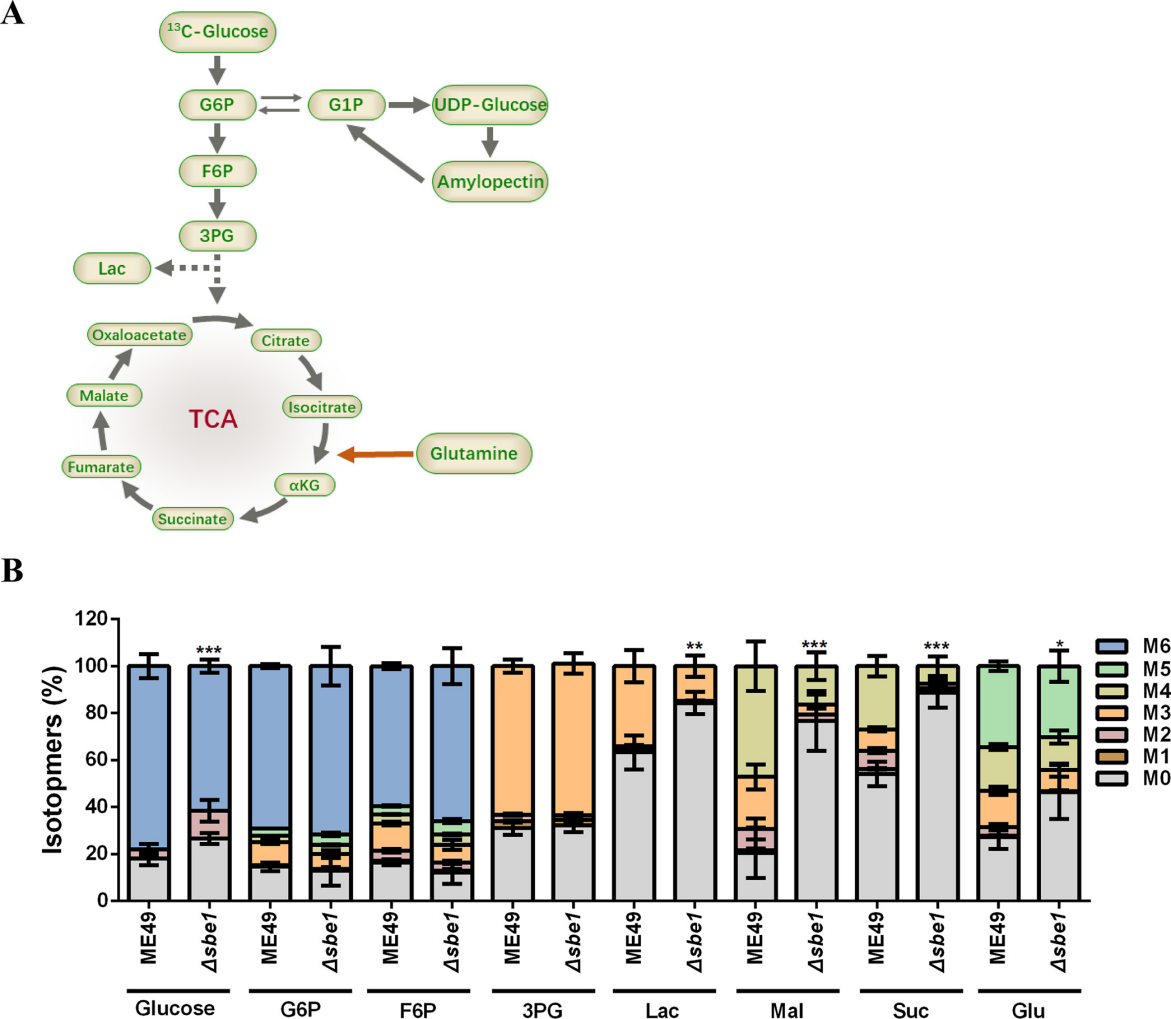
Flux of exogenous ^13^C_6_-glucose determined by metabolic flux. The tachyzoites of ME49 and *Δsbe1* strains were cultured in DMEM medium including 8 mM ^13^C_6_-glucose for 4 h. A. Schematic of ^13^C_6_-glucose labeling of intracellular metabolites. B. Flux of ^13^C_6_-glucose-derived tracer carbon into glycolysis and TCA cycle metabolites, and amino acids. G6P, 6-phosphate glucose; F6P, 6-phosphate fructose; 3PG, 3-phosphoglycerate; Lac, lactate; Mal, malic acid; Suc, succinic acid; Glu, glutamic acid; M0–M6 indicate the number of ^13^C-labeled carbons in the corresponding molecule. ***, P < 0.001; **, P < 0.01; *, P < 0.05, two-way ANOVA.

Glucose is the primary substrate of amylopectin synthesis in T. gondii (22). Since *SBE1* deletion caused amylopectin synthesis defects, we next examined whether SBE1 inactivation affected glucose metabolism. In the present study, fresh extracellular tachyzoites were labeled with ^13^C-glucose for 4 h, and the influx of ^13^C into the metabolic intermediates of glycolysis and the TCA cycle was analyzed (Fig. 3A–B). Compared with the ME49 strain, the inclusion of glucose-derived ^13^C into the glycolytic intermediate lactate and TCA cycle intermediates (such as malate and succinate) were decreased, indicating reduced flux through these two pathways. Unexpectedly, changes in other glycolytic intermediates (such as glucose-6-phosphate, fructose-6-phosphate, and 3-phosphoglycerate) were not as distinct, implying that other ways of supplementing glycolysis intermediates may exist. Furthermore, the influx of ^13^C-labeled glucose was significantly reduced in the *SBE1* deletion mutant, suggesting reduced flux of glucose into the parasite from the medium, which may also be responsible for affecting glycolysis and TCA metabolism. These results indicate that disruption of *SBE1* impairs the demand and utilization of exogenous glucose by tachyzoite, which may be more obviously in bradyzoites.

### Knockout of *SBE1* in *Δα-amy* mutant eliminates amylopectin accumulation but does not affect tachyzoite growth *in vitro* and virulence.

**FIG 4:**
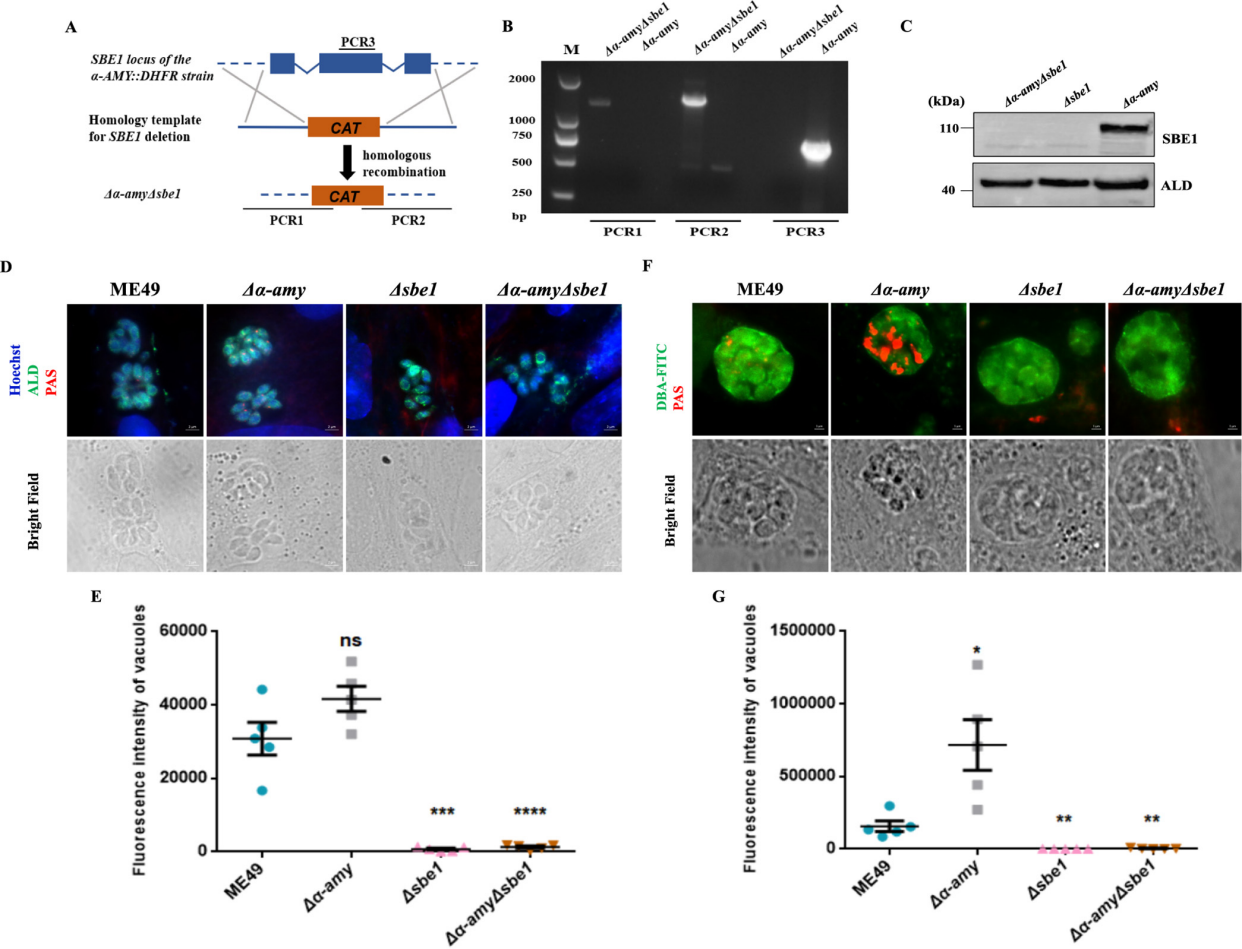
Generation and characterization of *Δα-amyΔsbe1* double-deletion mutant. A. Schematic illustration of generation of the *Δα-amyΔsbe1* mutant based on *Δα-amy* strain *via* CRISPR/CAS9-mediated technique. B. Diagnostic PCRs on a Δα-amy: DHFR*Δsbe1: CAT* clone. C. Western blotting confirmed disruption of SBE1 in *Δα-amyΔsbe1* double-deletion parasites. Parasite lysates were incubated with mouse anti-SBE1 antibody and rabbit anti-ALD served as the loading control. D. Amylopectin accumulation of tachyzoites was detected with PAS staining. E. Fluorescence intensity of PAS staining signals of tachyzoites. F. Amylopectin accumulation of bradyzoites was detected with PAS staining. G. Fluorescence intensity of PAS staining signals in Fig. 4E ****, P < 0.0001; ***, P < 0.001; **, P < 0.01; *, P < 0.05, ns represents not significant, Student's *t* test.

Based on our previous study (21), the loss of amylopectin degrading enzyme α-AMY caused abnormal amylopectin accumulation in bradyzoites, alleviated virulence, and decreased brain cyst formation. Furthermore, the absence of *SBE1* led to starch synthesis defects but did not affect virulence and brain cyst formation. To further probe whether amylopectin was the main element of phenotypic differences in tachyzoites, we disrupted *SBE1* co-expression with a *Δα-amy* mutant. We successfully obtained the *Δα-amyΔsbe1* mutant as determined via identification by PCR and Western blot (Fig. 4A–C). As shown in Fig. 4D and F, *SBE1* deletion eliminated the accumulation of amylopectin in the *Δα-amy* strain, which was not visible in both tachyzoites and bradyzoites of the *Δα-amyΔsbe1* mutant. In addition, the fluorescence intensity of PAS staining also affirmed that the amylopectin level in the *Δsbe1* and *Δα-amyΔsbe1* mutants was lower than that in the ME49 strain during the tachyzoite and bradyzoite stages, showing a significantly enhanced fluorescence signal in bradyzoites (Fig. 4E and 4G), which was consistent with previous research (21). The above results further confirmed that SBE1 plays a vital role in amylopectin synthesis.

**FIG 5:**
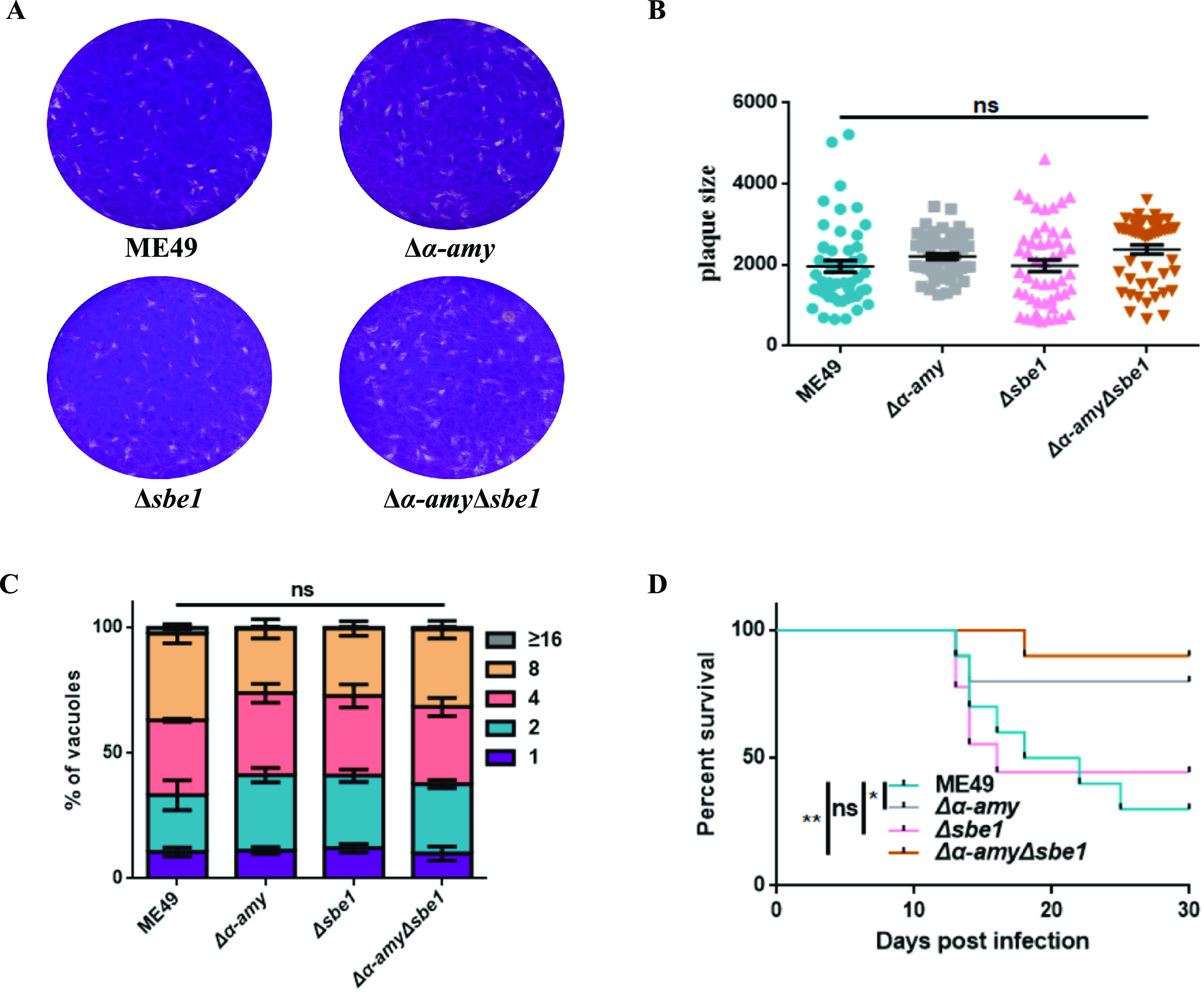
Growth and virulence of *Δα-amyΔsbe1* mutant *in vitro*. A. Plaque assay of parasites *in vitro*. Purified indicated tachyzoites were used to infect HFF cells, and 200 parasites were cultured for 14 days. B. Relative size of plaques in Fig. 5A C. Intracellular replication rates of parasites *in vitro*. Freshly egressed tachyzoites of indicated strains were used to invade HFF monolayer for 1 h, and then the parasites were grown for another 24 h. Parasite replication was determined in the same way as above. D. Survival curves of mice infected with tachyzoites of indicated strains. **, P < 0.01; *, P < 0.05, ns represents not significant, one-way ANOVA, two-way ANOVA, Mantel-Cox log-rank test. Data represent mean ± SEM of three independent experiments.

Next, tachyzoite growth *in vitro* and virulence were measured in each strain with amylopectin metabolism disorder. The results of both single and double knockout mutants revealed similar plaque sizes to the wild-type strain (Fig. 5A–B), and that of the replication ability further confirmed the robust growth of tachyzoites *in vitro* (Fig. 5C). From the virulence assay results, it could be observed that the virulence of both the *Δα-amy* mutant with defective amylopectin digestion and the *Δα-amyΔsbe1* mutant with defective amylopectin synthesis was attenuated in mice, which was different from the *Δsbe1* strain (Fig. 5D). These results illustrate that α-AMY was the determining factor in alleviative virulence, and changes in amylopectin metabolism were not directly relevant to parasite virulence.

### Inactivation of SBE1 impairs the intracellular replication of bradyzoites.

**FIG 6:**
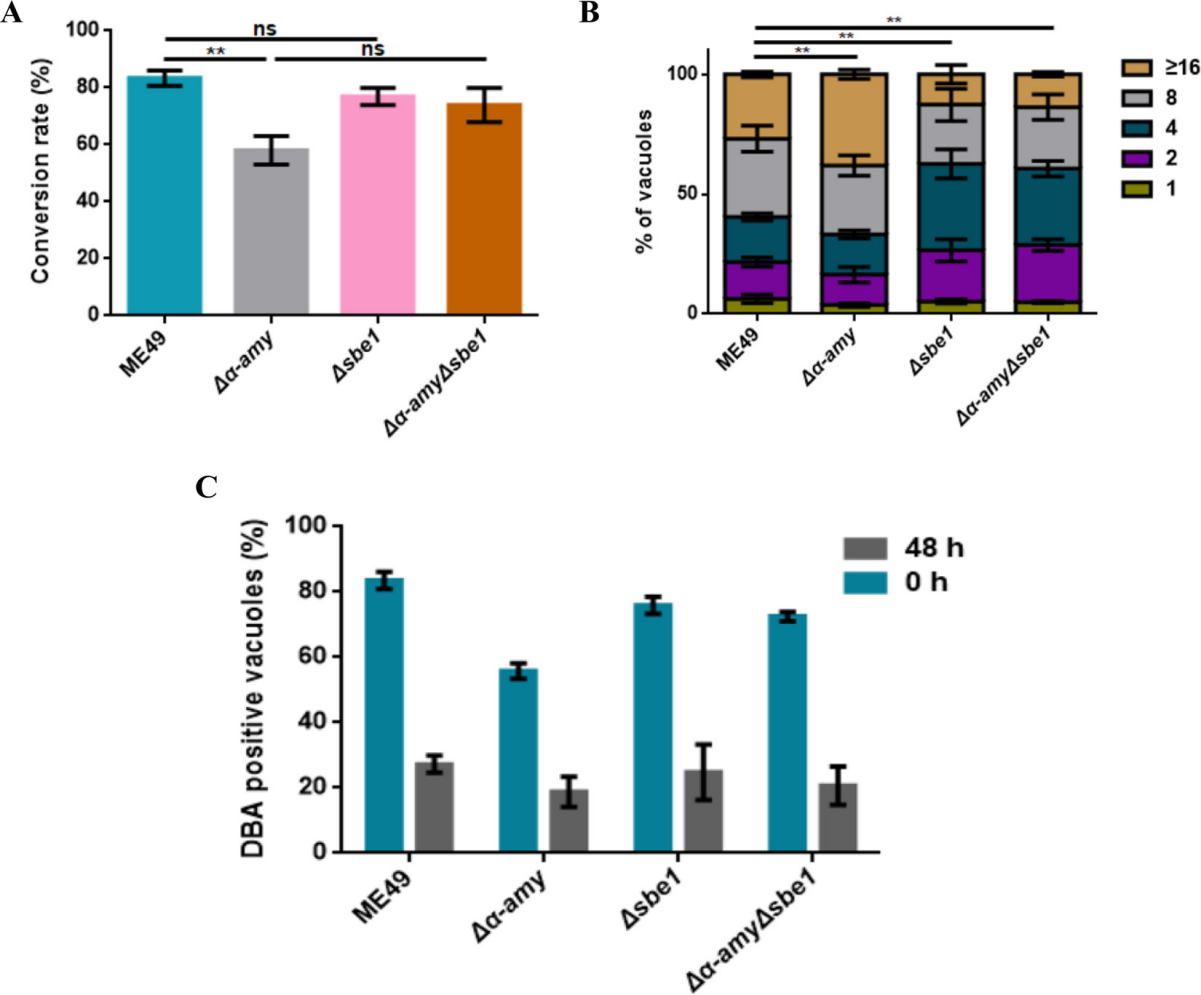
Starch branching enzyme 1 (SBE1) is important for replication of bradyzoites *in vitro*. A. Detection of the conversion rates of bradyzoites, statistics of the proportion of Dolichos biflorus *agglutinin* (DBA)-positive parasitophorous vacuoles (PVs) in all indicated parasites after 4 days of induction under alkaline culture conditions. B. Parasite replication under bradyzoite-inducing conditions. The indicated parasites were induced under alkaline culture conditions for 4 days and then grown under alkaline conditions for an additional 36 h. Parasite replication was determined in the same way as above. C. Detection of *SBE1* deletion mutant on the ability of bradyzoite reactivation to tachyzoites *in vitro*. The indicated strain was first cultured under alkaline culture conditions for 15 days to form bradyzoites (DBA-positive) and then changed to grow in tachyzoite culture conditions for 48 h. The samples were stained with DBA-FITC and a reduction of DBA-positive vacuoles means that bradyzoites were transformed into tachyzoites. **P < 0.01, ns represents not significant, one-way ANOVA, two-way ANOVA. Data represent mean ± SEM of three independent experiments.

Next, we investigated the possible function of amylopectin on bradyzoite growth and development. The conversion rate of bradyzoites was evaluated in *α-AMY* and *SBE1* deleted mutants. Compared to infection with the parental ME49 strain, infection with Δα-AMY resulted in a 25.3% reduction in bradyzoite conversion rate, while the bradyzoite conversion rate in the *Δsbe1* mutant showed no significant difference from that in the ME49 strain—in accordance with the brain cyst formation *in vivo* (Fig. 6A). Noteworthy, the conversion rate of bradyzoites was recovered in the *Δα-amyΔsbe1* mutant. Then, the intracellular replication of bradyzoites was implemented to examine bradyzoite proliferation. The replication of *α-AMY* deletion mutant was significantly increased with more parasitophorous vacuoles (PVs) containing eight parasites or more, while bradyzoites in the PVs of the *Δsbe1* and *Δα-amyΔsbe1* strains were almost less than four parasites, elucidating that *α-AMY* knockout resulting in starch accumulation in bradyzoites lead to enhanced intracellular replication of bradyzoites, whereas the proliferation of bradyzoites with amylopectin synthesis defect was significantly weakened (Fig. 6B). Based on previous studies speculating that amylopectin may provide energy for the conversion of bradyzoite to tachyzoite (13), we investigated whether defects in amylopectin metabolism would impact the reactivation process of bradyzoites. Each strain was induced in bradyzoites culture conditions for 15 days, and then the reactivation rate of bradyzoites to tachyzoites was monitored after 48 h in the tachyzoites culture conditions. As shown in Fig. 6C, the proportion of DBA-positive PVs in the *α-AMY* deficient mutant decreased from 55.75% to 17.89%, which was decreased by 51.1% and 51.84% in *Δsbe1* and *Δα-amyΔsbe1* mutants, respectively, with no noteworthy difference of proportion between the mutants and parent strain. These results indicated that the defects in amylopectin metabolism caused by *α-AMY* and *SBE1* knockout had no impact on bradyzoite reactivation *in vitro*. In short, all the results illustrated that SBE1 was crucial for intracellular replication of bradyzoites, though SBE1 might not contribute much to the development and reactivation of bradyzoites *in vitro*.

### SBE1 inactivation leads to abnormal and avirulent brain cyst formation.

**FIG 7:**
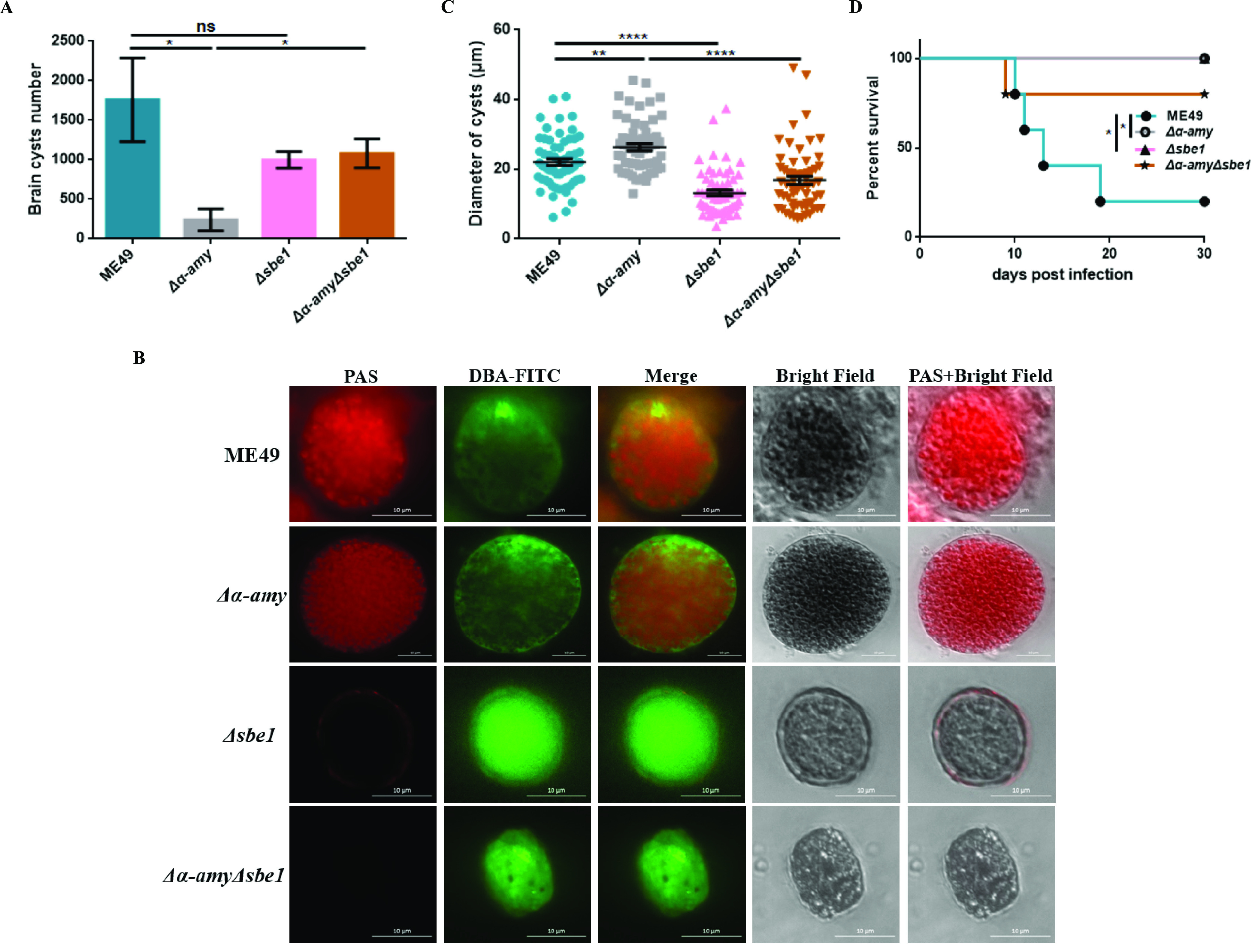
Effects of SBE1 deficiency on the morphology and function of cysts. A. Brain cysts of ICR mice infected with corresponding strains. The brain cysts of positive mice that survived 30 days after infection with the indicated strains were collected and stained with DBA-FITC, and the number of brain cysts was counted under a microscope. B. Detection of amylopectin accumulation in the brain cysts by IFA-PAS staining. The brain cysts were purified and then the amylopectin accumulation was detected with IFA-PAS staining. C. Relative sizes of brain cysts obtained in Fig. 7A. D. Brain cyst virulence of indicated strains. A total of 20 brain cysts were administered intragastrically per mouse (5 mice for ME49 and *Δα-amyΔsbe1* group, and 4 mice for *Δα-amy* and *Δsbe1* group), and the survival rate was monitored for 30 days. ****, P < 0.0001; ***, P < 0.001; **, P < 0.01; *, P < 0.05, one-way ANOVA, Mantel-Cox log-rank test.

To further verify the importance of amylopectin in chronic infection, ICR mice were infected with T. gondii and cysts were harvested for analysis. The number of brain cysts produced in starch synthesis-deficient mutants was not significantly different from that of the wild-type strains. Furthermore, the number of brain cysts in *Δα-amyΔsbe1* mutants was significantly restored compared to the *Δα-amy* strain (Fig. 7A). We also separated and purified cysts from brain tissue, and then performed IFA-PAS staining to detect the amylopectin accumulation in the purified cysts. Obvious starch accumulation staining could be observed in the ME49 cysts, and this signal was stronger in cysts lacking α-AMY. The entire *Δα-amy* cyst was filled with amylopectin granules, and cysts were significantly larger. As expected, *Δsbe1* and *Δα-amyΔsbe1* mutants were negative for PAS staining, consistent with the staining signals of bradyzoites induced *in vitro* (Fig. 7B). Subsequently, the diameter of the cyst was measured, and it was found that the absence of α-AMY would result in a noticeable increase of cyst diameter, while *SBE1* deletion caused cysts to become significantly smaller (Fig. 7C). The results of a cyst virulence assay also revealed that cysts with defects in amylopectin digestion and synthesis were avirulent to mice, despite the virulence of *Δsbe1* tachyzoites remaining robust, which also implied that these cysts were unable to achieve reactivation (Fig. 7D). In summary, these results showed that normal amylopectin metabolism is beneficial to develop normal cyst morphology and function, which is essential for the maintenance of chronic infections.

## DISCUSSION

Studies have shown bradyzoites and tachyzoites have different energy metabolisms, with bradyzoites characteristically having a massive amylopectin accumulation in the cytoplasm (23, 24). It has been hypothesized that this complex carbohydrate structure may play a key role in bradyzoite development and chronic infection maintenance. Recent research has implicated an amylopectin metabolism model and potential enzymes in T. gondii using plant and human glycogen metabolism as references (15). Until now, the physiological function of amylopectin, especially in bradyzoite development and conversion, has not been elucidated. Therefore, we clarified the biological function of SBE1 in the amylopectin synthesis of T. gondii and revealed the significance of amylopectin for tachyzoite growth and chronic infection reactivation. In addition, we speculated the role of the *SBE2* gene (*TGME49_209960*) encoding SBE in T. gondii infection in amylopectin synthesis and accumulation *in vitro* and *in vivo* as proof of concept. Future research still needs to investigate its expression and functions.

In this study, SBE1 enzyme activity was determined and SBE1 was found to bind and catalyze amylose, thereby reducing amylose and increasing amylopectin production (25). Deletion of *SBE1* did not affect tachyzoites growth under normal conditions. However, knockout of *SBE1* with deletion of *α-AMY*, which has been demonstrated to degrade amylopectin (21), caused the ablation of starch synthesis while tachyzoites still grew robustly *in vitro*. Therefore, it can be confirmed that *SBE1* was not necessary for normal tachyzoite functioning. Tachyzoites primarily utilize glucose and glutamine obtained from host cells for energy metabolism, and γ-aminobutyric acid can be accumulated rapidly in the extracellular environment which acts as short-term energy reserves to promote the motion and invasion of parasites. Furthermore, tachyzoites only accumulate starch under stress (11, 22, 26). Nevertheless, the virulence of both *α-AMY* deletion alone and in the double-knockout strain significantly receded, despite the amylopectin metabolism status being different between the two, which revealed that altered virulence was irrelevant to amylopectin metabolism. Furthermore, it has been reported that the absence of GP and amylo-α-1,6-glucosidase does not affect the growth and virulence of tachyzoites (14, 27). From these results, we conclude that amylopectin as an energy substrate has only minor contributions to the tachyzoite stage.

SS and SBE are considered the key enzymes required for amylopectin synthesis (17), and lack of *SS* in T. gondii leads to defects in amylopectin synthesis thus reducing the utilization and uptake of exogenous glucose (15). Our results indicated that deletion of *SBE1* also diminished the influx of glucose, thereby causing metabolic disorder. Obvious defects of tachyzoite replication ability were observed using labeled carbon, which suggested that amylopectin is a possible carbon source to provide energy under nutrition-deficient conditions. Our study also revealed that deletion of *α-AMY* resulted in visible amylopectin accumulation in the bradyzoites which enhanced replication, while loss of *SBE1* diminished replication. It has been controversial whether the altered formation of brain cysts between strains stems from amylopectin synthesis defects. Previous studies showed that brain cyst formation was significantly reduced in GP^S25E^ mutants while forming normally in *SS*-deleted mutants (14, 15). Our study showed that the accumulation of amylopectin resulted in the reduction of cyst formation, but lack of accumulation did not affect the formation of cysts. Moreover, the cysts from the *Δsbe1* mutant presented with abnormal morphology, which implied that the variation in cysts was indeed directly relevant to amylopectin. The above results also provided strong support for the perspective that amylopectin was essential for the formation and development of bradyzoites.

Amylopectin stored in *Eimeria* and *Cryptosporidium* oocysts impacts the infectivity and virulence of sporozoites (28, 29). Our cyst virulence results indicated that aberrant starch metabolism in cysts was nontoxic to mice, suggesting that normal regulation of amylopectin was critical for the functioning of cysts. Cysts with deficient starch synthesis resulted in insufficient energy supply during the conversion of bradyzoites to tachyzoites, leading to the failure of cysts to reactivate. Although bradyzoites with defective amylopectin synthesis could still be reactivated *in vitro*, we suspected that this phenomenon was caused by the different states of differentiation existing *in vivo* and *in vitro*. Thus, the culture conditions for bradyzoite development were insufficient to fully model animal infections, which appeared to be more hostile (30, 31). Since amylopectin is also observed in oocysts, it is necessary to further study the role of amylopectin metabolism in this life stage.

## MATERIALS AND METHODS

### Animals and parasites.

Male ICR mice (7-weeks old) were acquired from the Hubei Provincial Centre for Diseases Control and Prevention and co-housed under specific pathogen-free conditions at 25°C (Wuhan, China). All experimental procedures were approved by the Scientific Ethics Committee of Huazhong Agricultural University (HZAUMO-2020-0012), and all efforts were made to minimize the suffering of animals. Tachyzoites of T. gondii type II strain ME49 were propagated in human foreskin fibroblast cells (obtained from the ATCC, Manassas, VA, USA), cultured in DMEM medium supplemented with 2% FBS, and incubated at 37°C and 5% CO_2_.

### Phylogenetic analysis.

The protein sequence of *Toxoplasma* SBE1 was compared on the UniProt database (https://www.uniprot.org/). Sequences of representative species (such as animals, plants, bacteria, and other apicomplexan parasites) with high homology to T. gondii SBE1 (*Tg*SBE1) were selected. Then, the protein sequences were downloaded for each species, and Clustal W v1.2.2 was used to perform multiple sequence alignment and remove low-homology regions. The phylogenetic tree analysis was performed using MEGA v6.0, and the Jones-Taylor-Thornton (JTT) model was applied to construct a neighbor-joining method phylogenetic tree. The Bootstrap value was set to 1,000 for construction, and finally, the phylogenetic tree was drawn according to the scale.

### Recombinant protein SBE1 enzymatic activity assay.

Recombinant protein SBE1 enzymatic activity assay was detected according to previous research (32, 33). Briefly, 10 μL recombinant protein was incubated with different concentrations of amylose (10 to 1,500 μg/mL) in 180 μL 50 mM NaCl in PBS buffer (pH = 7.0) at 37°C for 30 min. Subsequently, 10 μL iodine solution was added to the reaction mixture, and heat-inactivated protein was used as the control group. After the reaction finished, the absorbance was obtained at 660 nm, and the kinetics of enzyme-catalyzed reactions, including *K*_m_ and *V*_max_ values was calculated by Prism v6.0 (GraphPad Software, La Jolla, CA, USA). One unit of enzymatic activity was defined as a decrease in absorbance of 1.0%/min at 660 nm.

### Generation of transgenic strains and phenotypic analysis.

The CRISPR/CAS9-mediated homologous system was used for *SBE1* (*TGME49_316520*) knockout in a T. gondii ME49 strain and to construct the *Δsbe1* mutant, as described previously (34, 35). Briefly, the pSAG1‐Cas9‐sgUPRT plasmid and 5H-DHFR-3H homologous templates with drug screen labels based on the *SBE1* gene were co-transfected into purified tachyzoites and then screened with 1 μM pyrimethamine or 30 μM chloramphenicol. Positive single clones were identified by diagnostic PCRs. In addition, the pα-SBE1::α-AMY-CAT fragment and CRISPR plasmid based on the *hxgprt* locus were used to transfect into the *Δsbe1* mutant and selected with 30 μM chloramphenicol to construct the complementation strain (Comp-SBE1), and positive clones were identified by diagnostic PCRs. All primers and plasmids used in this study are listed in Table S1 in the supplemental material.

### Western blot analysis.

A 6×His-tagged polypeptide corresponding to the SBE1 fragment was expressed and purified from E. coli to generate antibodies against SBE1. The recombinant protein was used to immunize mice to acquire polyclonal antibodies. The total protein was extracted according to the manufacturer’s protocols, and proteins of about 4 × 10^7^ parasites were separated by 12% SDS-PAGE prior to transferring to PVDF membranes (Millipore, MA, USA), and then the PVDF membranes were blocked in 2% BSA at room temperature for 2 h. The membranes were incubated with rabbit anti-*Tg*ALD (provided by Dr David Sibley, Washington University in St. Louis) and mouse anti-*Tg*SBE1 at 4°C overnight. Subsequently, the membranes were detected with secondary antibodies (1:2000 dilutions; Beyotime, Shanghai, China) at room temperature for 1 h after washing with TBST five times. Eventually, the protein signal was detected with an ECL Plus Western Blotting Detection System (Image Quant LAS 4000mini, United States).

### Plaque assays.

Freshly egressed tachyzoites were purified and added (200 parasites per well) to six‐well plates pre‐seeded with HFF monolayers, and then were grown for 14 days at 37°C with 5% CO_2_. Subsequently, the cells were fixed with 4% paraformaldehyde, stained with crystal violet, and imaged on a scanner to analyze the relative size of plaques, as described previously (Shen & Sibley, 2014).

### Intracellular replication assay.

The method of intracellular replication assay was performed as previously described (36). Briefly, freshly harvested tachyzoites were purified by filtration through 3 mm polycarbonate membranes and then inoculated on coverslips 1 h. Non-invaded tachyzoites were washed away, and the remaining ones continued to be cultivated for 24 h. Swine anti-Toxoplasma IgG was used to stain extracellular parasites, and rabbit anti-TgALD was responsible for all parasites. After staining with secondary antibodies Alexa Fluor 594-conjugated goat antirabbit IgG and FITC conjugated goat anti-swine IgG, respectively (Life Technologies, Camarillo, CA, United States), the extracellular parasites stained both red and green were not counted.

### Bradyzoite differentiation *in vitro*.

The method of inducing bradyzoite differentiation *in vitro* was performed as described previously (37). Briefly, ME49 stain tachyzoites were forced to egress by needle passages and allowed to invade HFF monolayers seeded in T25 flasks. After 1 h under standard growth conditions, the non-invaded extracellular parasites were washed off, then the parasites were sequentially cultured in T25 flasks under bradyzoite-inducing conditions (RPMI 1640 medium supplemented with 50 mM HEPES and 1% fetal bovine serum, PH 8.2, ambient CO_2_) for 4 days.

### Immunostaining-compatible Periodic acid-Schiff (PAS) staining.

The method of immunostaining-compatible PAS staining was performed as previously described (14, 20). Briefly, purified tachyzoites and bradyzoites were added to infect fresh HFF cells seeded on coverslips. To stain tachyzoites, the parasites were cultured under standard culture conditions for 24 h. To stain bradyzoites, the intracellular parasites were cultured with 1% FBS under alkaline culture conditions for 4 days. All samples were fixed with 4% paraformaldehyde at 25°C for 15 min, permeabilized with 0.1% Triton X-100 at 25°C for 15 min, and 10% FBS was used to block at 37°C for 2 h. Next, rabbit anti-ALD was incubated for tachyzoites staining at 37°C for 20 min, and Dolichos biflorus agglutinin (DBA)-FITC was incubated for bradyzoites staining. Subsequently, the samples were stained with 1% periodic acid (Sigma-Aldrich, St. Louis, MO, USA) at 25°C for 5 min and washed five times with PBS. Then samples were incubated with Schiff's reagent (Sigma-Aldrich) for 15 min and washed five times with PBS. After staining, the PAS staining was determined using an Olympus BX53 fluorescence microscope (Olympus, Tokyo, Japan). For amylopectin quantification, the PAS fluorescence intensity was measured and analyzed using ImageJ v1.57 (https://imagej.nih.gov/ij/) by obtaining the mean fluorescence intensity as previously described (14).

### Metabolic labeling.

The ^13^C-labeled glucose metabolic flux of T. gondii was detected as described in previous research (15). Briefly, freshly purified tachyzoites of indicated strain were cultured under standard conditions for 3 days, and then 2 × 10^6^ extracellular parasites were collected and washed with glucose-free medium three times, followed by continuing to culture in a medium containing 8 mM ^13^C_6_-glucose at 37°C for an additional 4 h. Finally, all the samples were harvested for the next analysis of metabolic flux by ultra-high-performance liquid chromatography high-resolution mass spectrometry (UHPLC-HRMS).

### Virulence tests and brain cysts formation in mice.

Female ICR mice (8-weeks old) were intraperitoneally infected with 100 freshly egressed tachyzoites each or orally infected with 20 brain cysts/mouse of indicated strains. All mice were monitored daily to assess mortality for 30 days, and the serum samples of the surviving mice were collected for soluble *Toxoplasma* antigen (TSA)-based ELISA to eliminated uninfected mice after 30 days postinfection. For counting of brain cysts, the anti-TSA negative mice were eliminated and sacrificed to isolate the brain tissues, which were homogenized to measure the number of *Toxoplasma* cysts by DBA-FITC staining, as described previously (38).

### Statistical analysis.

All statistical analyses were performed with Prism v6.0 and presented as the mean ± SEM. The statistical differences between groups were determined using Student's *t* test, one‐, or two‐way analysis of variance as indicated in the figure legends. A value of *P < *0.05 was defined as statistically significant. Cumulative mortality was graphed as Kaplan-Meier survival plots and analyzed by Mantel-Cox log-rank test.
