# Supplementary material for: Starch Branching Enzyme 1 Is Important for Amylopectin Synthesis and Cyst Reactivation in Toxoplasma gondii
Source: Microbiol Spectr. 2022 Apr 21;10(3):e01891-21. doi: 10.1128/spectrum.01891-21 (PMC9241709; doi:10.1128/spectrum.01891-21)
Supplement: SUPPLEMENTAL FILE 1 — Supplemental material. Download spectrum.01891-21-s001.pdf, PDF file, 1.5 MB [file spectrum.01891-21-s001.pdf]

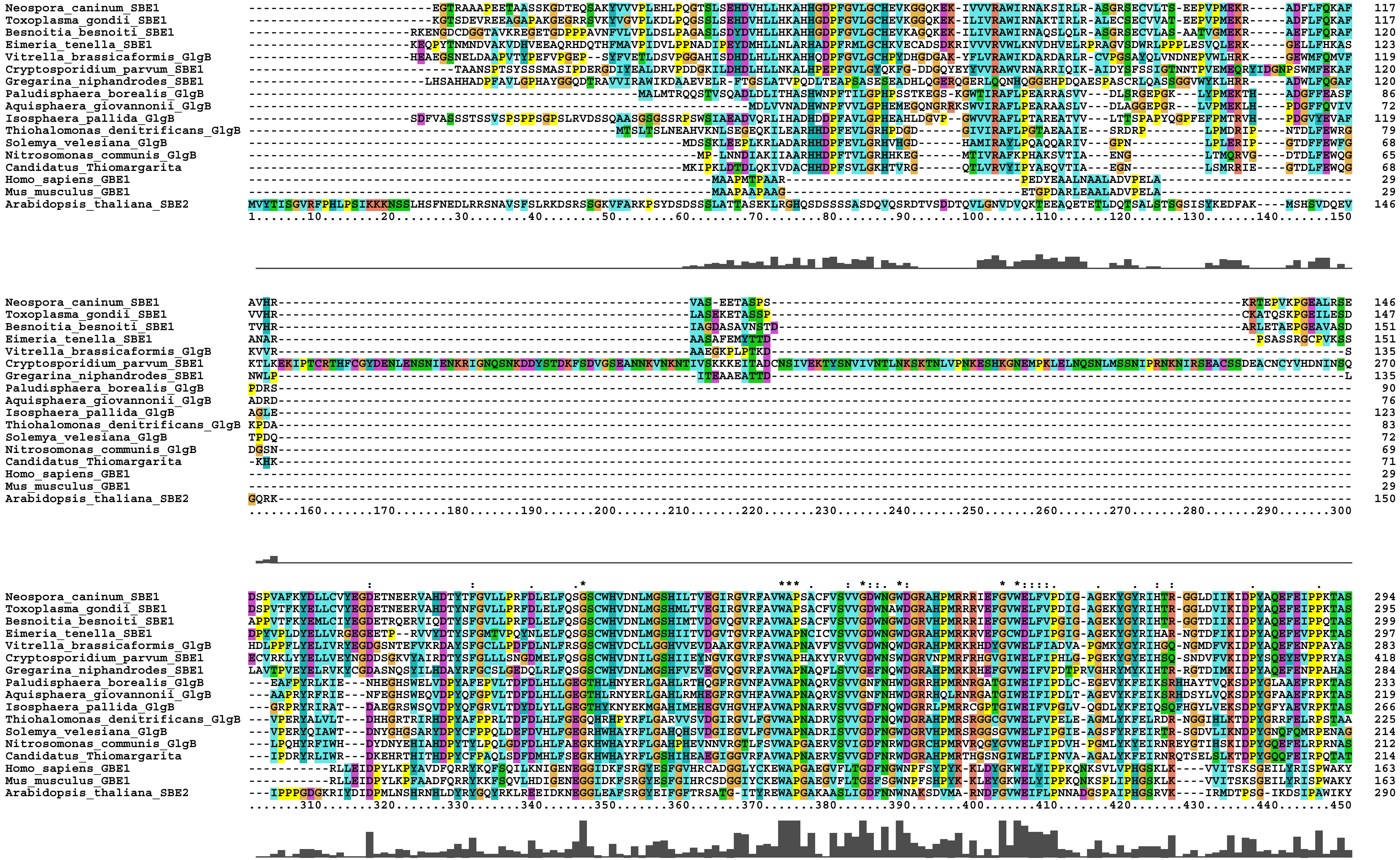
Neospora_caninum_SBE1
Toxoplasma_gondii_SBE1
Besnoitia_besnoiti_SBE1
Eimeria_tenella_SBE1
Vitrella_brassicaformis_GlgB
Cryptosporidium_parvum_SBE1
Gregarina_niphandrodes_SBE1
Paludisphaera_borealis_GlgB
Aquisphaera_giovannonii_GlgB
Isosphaera_pallida_GlgB
Thiohalomonas_denitrificans_GlgB
Solemya_velesiana_GlgB
Nitrosomonas_communis_GlgB
Candidatus_Thiomargarita
Homo_sapiens_GBE1
Mus_musculus_GBE1
Arabidopsis_thaliana_SBE2

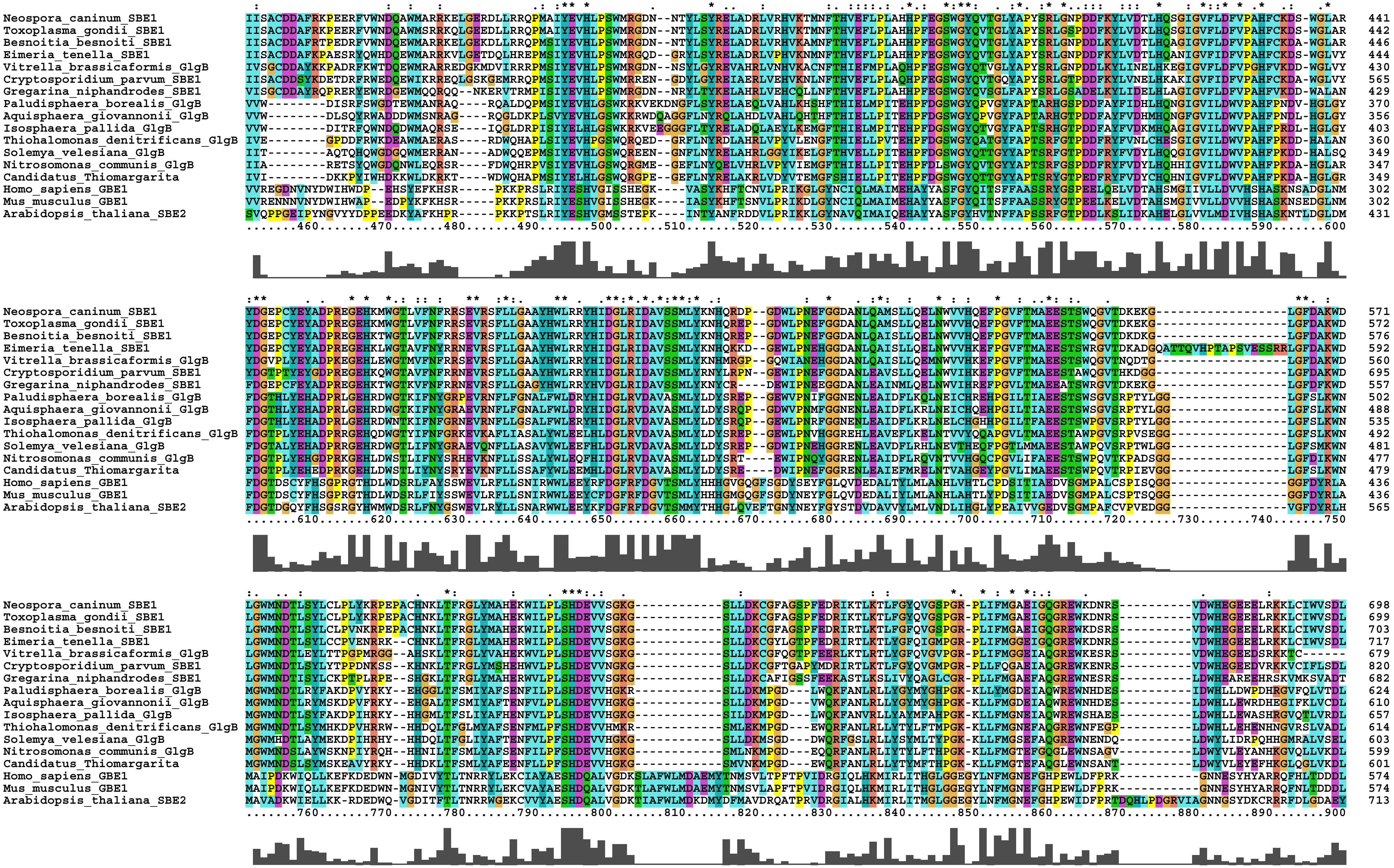
Neospora_caninum_SBE1
Toxoplasma_gondii_SBE1
Besnoitia_besnoiti_SBE1
Eimeria_tenella_SBE1
Vitrella_brassicaformis_GlgB
Cryptosporidium_parvum_SBE1
Gregarina_niphandrodes_SBE1
Paludisphaera_borealis_GlgB
Aquisphaera_giovannonii_GlgB
Isosphaera_pallida_GlgB
Thiohalomonas_denitrificans_GlgB
Solemya_velesiana_GlgB
Nitrosomonas_communis_GlgB
Candidatus_Thiomargarita
Homo_sapiens_GBE1
Mus_musculus_GBE1
Arabidopsis_thaliana_SBE2

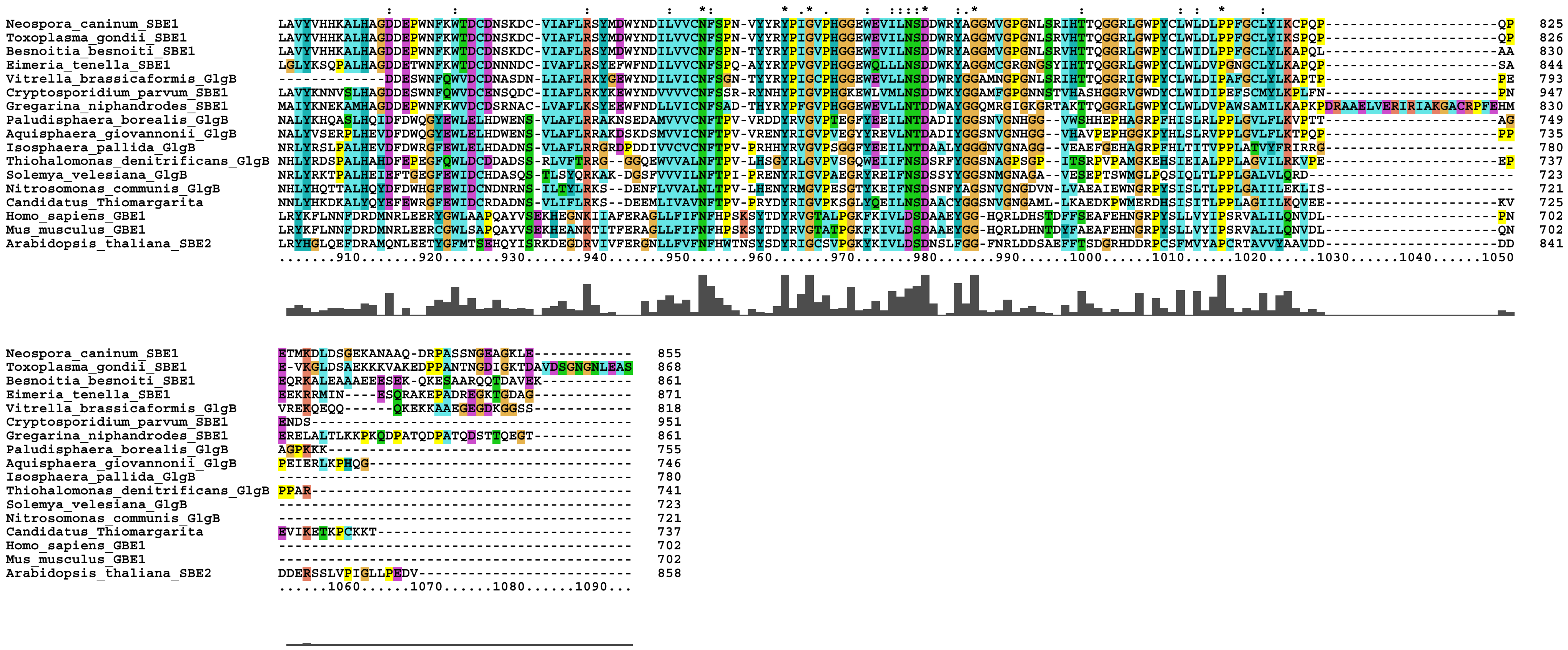

Fig. S1. Alignment of amino acid sequence and phylogenetic relationships analysis of starch branching enzyme in T. gondii and selected species.

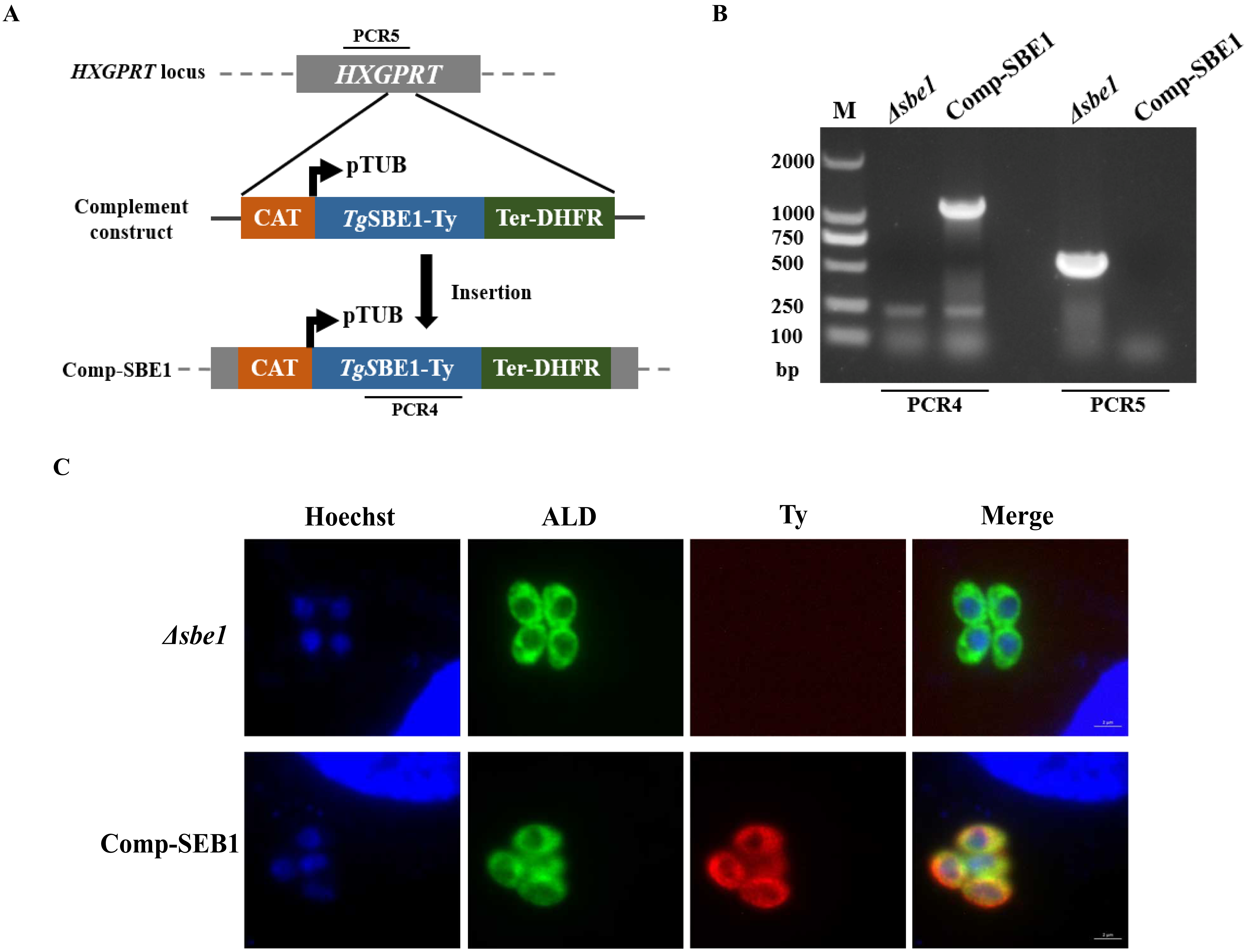

Fig. S2. Construction of SBE1 complementation strains.
A. Schematic illustration of generation of the Comp-SBE1 strain via CRISPR/CAS9-mediated technique.
B. Diagnostic PCRs on a Comp-SBE1 clone. C. The presence of SBE1 was confirmed by IFA. Samples were stained with mouse anti-Ty and rabbit anti-*T. gondii* ALD.

Table S1: Primers used in this study.

Primer	Sequence (5’-3’)	Used for
gRNA-*SBE1*-Fw	GGGTCTGCATATTCATAGCAGTTTTAGAGCTAGAAATAGC TAGAAATAGC	Construction of gRNA plasmid for *SBE1* knockout
gRNA-Rv	AACTTGACATCCCCATTTAC	
pUC19-Fw	GGCGTAATCATGGTCATAGC	Amplification of pUC19 vector
pUC19-Rv	ACTGGCCGTCGTTTTACAAC	
loxp-DHFR*-loxp-Fw	CAACCCGCGCAGAAGACATC	Amplification of loxp-DHFR*-loxp fragment
loxp-DHFR*-loxp-Rv	GGACACGCTGAACTTGTGGC	
U5-*SBE1*-Fw	GTTGTAAAACGACGGCCAGTCTCTCATGCACTCAACATC	Amplification of the 5’ homologous arm of *SBE1*
U5-*SBE1*-Rv	GATGTCTTCTGCGCGGGTTGCTTCTCCTGACTGAGGTGAC	
U3-*SBE1*-Fw	GCCACAAGTTCAGCGTGTCCGTGAAGGCGCGTGAAAACCC	Amplification of the 3’ homologous arm of *SBE1*
U3-*SBE1*-Rv	GCTATGACCATGATTACGCCCGTACTCCTCCATCTTGGC	
5’-UpU5-*SBE1*	GGTAGCCCGATATTCACATGCAG	Verification of *SBE1*deletion by PCR1
3’-In DHFR*	GATTTGTGAGGACGACTCAC	
5’-In DHFR*	CACGACAGCAGACAACTTTC	Verification of *SBE1* deletion by PCR2
3’-DnU3-*SBE1*	GTGCCGTGCGAAAAACACGGAAC	
In-*SBE1*-Fw	GTCACAGGTTTATACGCGCC	Verification of *SBE1*

In-*SBE1*-Rv	GCTCTTGGAGGAGAGACATG	deletion by PCR3
Vec-Fw	GAGGTCCACACGAACCAGGAC	Amplification of the complementation vector
Vec-Rv	CTTGCTCACCATTTTAGATC	
SBE1-CDS-Fw	GATCTAAAATGGTGAGCAAGATGGGAGATCTTCGAGCATC	Amplification of *SBE1* CDS
SBE1-CDS-Rv	GTCCTGGTTCGTGTGGACCTCCTCCTGGCGCTCGTAAACAG	
gRNA-*HXGPRT*-Fw	GCAGAACTTACTTCGGCGAGGGTTTTAGAGCTAGAAATAGC	Construction of gRNA plasmid for *HXGPRT* knockout
PCR4-*SBE1*-Fw	GTGGACATGGCCGCTGCATG	Verification of *SBE1* complementation by PCR4
PCR4-*SBE1*-Rv	GTTGTTGTCGCCACGCATCCAG	
In-*HXGPRT*-Fw	CAAGGGCCGTATTGAGCCCATG	Verification of *HXGPRT* deletion by PCR5
In-*HXGPRT*-Rv	CACACGCCTTCCAAATGTCGCG	
CAT-Fw	CGTGTTTACTCGGATACTGACGAGGTCGACGGTATCGATAAGC	Amplification of CAT fragment
CAT-Rv	CCCTGCCACTGTTTATTCGCGCTCTAGAACTAGTGGATCG	
p-*SBE1*-CAT-U5-Fw	GATGCTACACATTTCTCACC	Amplification of the 5’ homologous arm of *SBE1* based on CAT tag
p-*SBE1*-CAT-U5-Rv	GTCAGTATCCGAGTAAACACG	

p-*SBE1*-CAT-U3-Fw	CGAATAAACAGTGGCAGGG	Amplification of the 3' homologous arm of *SBE1* based on CAT tag
p-*SBE1*-CAT-U3-Rv	AGTTGCTAGCTCTACTTCGC	
3'-In CAT	GCACTGTGAGCACTGATAGG	Verification of *SBE1* deletion by PCR1
5'-In CAT	GGAGTGTTTCGCAGCAAGC	Verification of *SBE1* deletion by PCR2
